# Experiences of Next-of-Kin to Foreign-Born Dying Patients Cared for in Specialist Palliative Care: A Qualitative Study

**DOI:** 10.1177/00302228231224000

**Published:** 2023-12-21

**Authors:** Maria E. Carlsson, Katarina Hjelm

**Affiliations:** 1 Department of Public Health and Caring Sciences, 174463 Uppsala University, Uppsala, Sweden

**Keywords:** next-of-kin, foreign-born patients, specialized palliative care, experiences, qualitative study

## Abstract

Earlier studies have shown that healthcare personnel in specialized palliative care see patients with migrant backgrounds as others and that they, as providers, are unable to provide culturally competent care. Thus, these studies indicate a taken for granted perception, instead of knowledge based on experiences or scientific knowledge. The objectives of this study were to explore preferences, expectations on and experiences of specialist palliative care from next-of-kin of migrants of different origin. This study used a qualitative methodology, and the data were analyzed with systematic text condensation. The interviews were based on semi-structured interviews with seven next-of-kin. Four code groups represented the next-of kin’s experiences: *The wishes and needs of the foreign-born person in a palliative phase form the care; The families’ prerequisites for, and the impact of, palliative informal caregiving; The staff working in accordance with a palliative care approach; Palliative care in a seamless care chain.*

## Introduction

In 2015, the number of migrants was the highest ever, as there was an influx of refugees to Sweden (SCB Statistics Sweden, 2016; Statistiska Centralbyrån, 2022). The new arrivals mainly originated from Afghanistan, North Africa, and Syria, and they were added to an already heterogeneous migrant population with persons from Scandinavia, Southern Europe, Latin America, Asia, the Middle East, and Africa, forming a multilingual group with great dissimilarities in language and cultural backgrounds. At the same time as there were new demands posed on healthcare from the newcomers, the situation in Sweden was that of an increasing aging population. Thus, there is a need to decrease costs from residential care to home care, which often requires support from relatives.

Studies have shown that the majority of patients and the general public prefer to die at home, given the choice (Coupland et al., 2011; Gomes et al., 2013). Many migrants in the palliative stage are cared for in their homes with the help of their relatives (de Graff et al., 2010; Shabnam et al., 2019). Healthcare providers often assume that migrant families care for the patients and they, therefore, did not need as much professional support as non-migrant families (Paal & Bűkki, 2017). In studies focusing on what migrants from Asia, Morocco, and Turkey say about where they want to be cared for, it was shown that relatives often felt an obligation to care for their next-of-kin in their homes (de Graaff et al., 2010; 2012; Heidenreich et al., 2014; Kiwi et al., 2018; Shabnam et al., 2019; Worth, 2009). Caring for a severely ill person in the home poses demands in order to fulfill the basic needs of care, in particular hygienic aspects and medication delivery; thus, the need for knowledge about these issues is important. To cease home care might be related to exhaustion, both physically and mentally (Kiwi et al., 2018; Shabnam et al. (2019). The migrant family member caring for a relative in the home, thus, might be trapped in a situation with particularly high demands and low control, which increases stress as many of them may deal with limited or broken social networks, have language difficulties, and limited economic resources (Heidenreich et al., 2014). Studies have also shown there is a lack of knowledge among migrant families about which palliative care services are available (Shabnam et al., 2019; Worth, 2009) and that the lack of culturally equivalent words for palliative care and hospice complicated this issue further (Shabnam et al., 2019). However, similarly, Paal and Bűkki’s (2017) study showed a limited understanding and knowledge of palliative and hospice care, although this was seen in both groups (both migrants and German natives).

In a qualitative study, the question was posed whether the cornerstones of palliative care, that is to improve quality of life and relieve suffering, as prolonging life is not an objective anymore, are congruent with the perspectives of immigrant families with a Turkish or Moroccan background living in the Netherlands (de Graaff et al., 2010). The main concerns expressed about good care were maximum treatment and curative care until the end of their lives, never having hope taken away, devoted care by their families, avoiding shameful situations, and dying with a clear mind. As many of them are holding out for a cure till the end of life, they find good palliative care to be a contradiction. The diagnosis, prognosis, and end-of-life (EOL) decisions were seldom discussed with the patient, and communication about pain or mental problems was often limited. Their perspectives conflict with the dominant principles of palliative care, for example, the emphasis on quality of life and advanced care planning, including discussions of diagnosis and prognosis with the patient and the family.

In a Swedish study, healthcare professionals’ understandings of cross-cultural interaction during end-of-life care were investigated through focus group interviews (Milberg et al., 2016). Even though they had limited experience of cross-cultural interaction in end-of-life care, they talked about it as specific challenges. These challenges included communication barriers; unusual emotional and pain expressions; and they expected that the patient’s families would be different and that the patients and their families lack knowledge. The core of these encounters was described as meeting “the unknown”. Further, the staff talked about patients whose backgrounds they did not share in a homogenizing manner and did not seem confident enough to be able to provide good quality care. Patients with migrant backgrounds were seen as others, and they viewed themselves as providers who were unable to provide culturally competent care. This was based on perceptions that the existence of a difference and uncertainty was a given expectation when caring for patients with migrant backgrounds (Karlsson, 2008; Torres et al., 2016). Thus, these studies indicate a taken for granted perception, instead of knowledge based on experiences or scientific knowledge.

The conclusion of Andershed’s (2008) comprehensive literature review on relatives’ situation in palliative care was that healthcare staff have a large responsibility so that the relatives experience “involvement in the light.” To accomplish good palliative care of a dying patient, especially in their own home, the communication between the healthcare staff and the family is vital. How this actually functions when the dying patient is foreign-born, to our best knowledge, has not been investigated. Even though individuals who do not speak the mother tongue of the host country are often excluded from studies, it constitutes a risk for inequitable palliative care. In spite of this, even when foreign-born individuals participate in studies, the analysis is not always conducted with a cultural perspective (Henoch et al., 2016). The research question in this study focuses on preferences, expectations, and experiences of specialist palliative care from the next-of-kin of migrants of different origin.

## Material and Methods

### The Settings and Participants

In Sweden, specialized palliative care is meant for patients with life-threatening diseases with complex palliative care needs that require attention from professionals with specialized palliative care skills and knowledge (Lundström et al., 2012; Radbruch & Payne, 2009). The organizations that provide such care are specialized palliative care teams, hospices, and palliative care units in hospitals. In the Swedish welfare system, there is a benefit for closely-related persons when caring for patients. Specifically, one can receive money when caring for a close relative in a life-threatening condition when losing work-related income. Both the sick person and the relatives that care for the person must be insured in Sweden, and the time-frame is 100 days (Forsakringskassan.se). The central aspects of ‘Specialized advanced palliative home care’ (AHC) are as follows:

- Provides palliative care
- Physician led
- Team-based
- Qualified medical technology is available in the home
- Provides a service on a 24-h basis
- Immediate availability of a hospital bed if necessary

The Advanced Home Care team (AHC) in the present study mainly provides medical care and support. If the patient and caregiver need help with activities of daily living (ADL), they get help from the local municipality Home Service (HS). The AHC team receives support, if required, from the local hospice in the city that will admit patients on short notice.

Participants were recruited from spring 2020 to spring 2023. The researchers approached the healthcare staff from one specialized palliative home care team to identify eligible next-of-kin to patients that have died three to twelve months earlier, in order to not disturb the bereavement process and to decrease the influence of recall bias. The next-of-kin were contacted via telephone by the staff that cared for their deceased family member; thereafter, they were given information about the study and assured that data would be handled by a specialized nurse and a researcher who not involved in the patient’s care (MC). If they expressed an interest in taking part, an information sheet about the study and a form with informed consent were sent by mail. If necessary there was a possibility to get an interpreter. However, all spoke fluent Swedish. Following informed consent, contact was made with the next-of-kin, and the times and places for the interviews were decided together with the participants. A nurse specialized in palliative care (the first author) performed the interviews individually, either face-to-face or by phone. The interviews were 19–83 minutes long. All interviews were audio-recorded. The researcher used a semi-structured interview guide (see Appendix A) during the interview, opening for informants’ thoughts, while at the same time staying close to certain topics designed by the researchers (Patton, 2015). The seven interviews generated very rich data.

The sample comprised one husband, one male in a living apart relationship, four daughters, and one son to patients that were born in Hungary, Iran, Kurdistan, Bosnia, Chile, and Lebanon. Two respondents were sisters. All the foreign-born persons (patients) had migrated to Sweden approximately 25–47 years ago.

### Analysis of Data

All interviews were transcribed by the first author in a slightly modified verbatim mode (Malterud, 2014), and the analysis was conducted by systematic text condensation inspired by Giorgi’s phenomenological approach and described by Malterud (2012). The analysis followed four steps: (1) reading all material to gain an overall impression and creation of preliminary themes, (2) identifying and coding units of meaning that represented various aspects of the families’ experiences of palliative care, (3) summarizing and condensing the content of each subgroup, and (4) synthesizing the results through re-contextualization (Malterud, 2012). Data are presented as descriptive summaries with code groups and subgroups, illustrated with quotations. The researchers regularly discussed the emerging analysis, enhancing the trustworthiness of the findings.

### Ethics

The study was implemented in accordance with the Helsinki Declaration and with written informed consent (Codex, 2023). In case negative or strong reactions were aroused during the interviews, a referral was made to the social worker in the palliative care team. This study was approved by the Swedish Ethical Review Authority (EPN D-number 2019–02,971).

## Findings

The analysis process yielded four code groups and eleven subsequent sub-codes, describing the families’ meeting with the specialized palliative care (Table 1.)

**Table 1.** Code Groups and Sub-codes.

Code groups	Sub-codes
The wishes and needs of the foreign-born person in a palliative phase form the care	Wishes and needs
	Communication and awareness
	Culture/religion
The families’ prerequisites for, and the impact of, informal, palliative caregiving	Social situation
	Emotions
The staff working in accordance with a palliative care approach	Social support
	Medical treatment and accessibility
	Competence and attitude
Palliative care in a seamless care chain	Hospital care
	Specialized palliative homecare
	Hospice care

### The wishes and Needs of the Foreign-Born Person in a Palliative Phase Form the Care

The common theme in all interviews was that the deceased person’s wishes and needs were the superior principle in all the decisions made about their care, both regarding communication aspects and pertaining to cultural or religious issues.

#### Wishes and Needs

In all families, there have not been any discussions about other care-forms than specialized palliative home care at first, because that was the wish of the deceased person. However, when the situation deteriorated and the deceased person wished for hospice care instead, the transfer happened very fast, within one day.

My wife didn't want to go anywhere; she wanted to die here in the house. This is our dream, so she had made up her mind. I let her decide. (Informant 4)

I got a bit upset. Then, she said that I have to think about what she mainly wants to do (move to hospice). (Informant 7)

Even aspects of who should care for the deceased person and what should be done were dependent on the deceased person’s wish or need. The first quotation illustrates the deceased person’s strong will. The last quotation reflects a strong feeling that only a specific next-of-kin should do all the caring and/or nursing.

He didn't want help from outsiders, uh, not from good friends, like his wife or from my mother, not from my siblings, but only from me. (Informant 2)

She was very concerned about her own privacy; so, the fact that I could be with her was incredibly important in order to somehow limit the number of people. (Informant 6)

#### Communication and Awareness

In two families, communication between the staff and the deceased person who needed an interpreter was an issue because of the language barrier; however, in both these families, the husbands needed even more help with interpretation by the informant than the deceased person.

She wanted to be cared for at home; her concerns were the language...what will the care look like and how will she be able to explain herself to communicate everything medically and so on, but it went better than we thought. (Informant 5)

Dad is a doctor; of course, he wanted information and, as a doctor, he had questions and so he asked them and wanted me to translate them. (Informant 6)

Communication problems could also arise because of the deceased person’s medical condition, illustrated in the quote below. In the other families, the deceased person spoke fluent Swedish and had no communication problems with the staff.

He had problems but not because of any language barriers, but because of the tumor in his lungs. So, it was physically difficult; so, he was whispering a lot...he was so exhausted. He was so sick that everything was tiring; talking, everything like that. He had difficulty to talk. (Informant 2)

All informants had perceived that the deceased person was fully aware of their situation, but the families did not talk openly about that or their feelings in front of the impending death.

She understood afterwards, when she didn't continue with any treatment, then she understood. We also understood without saying anything...she never brought it up.... like when I die, are you going to, or I asked him several times, are you telling the truth, did she say anything and he said no, no she hasn't mentioned anything; we never mentioned it. (Informant 5)

#### Culture/Religion

The deceased persons and their families had very few wishes about cultural or religious issues. The most common wish was that the deceased persons did not want to get help from male staff with personal hygiene, which the informant discussed below.

I mention it as a feeling how important it is for a person who comes from another culture. In some cultures, it is, but it's also a matter of personal choice because mom was incredibly concerned about her dignity. This nudity thing, it's hard because maybe we don't have this natural (laughter) attitude towards it that way or that we think it's a bit intimate and then I know that my mother needed it to not be male nurses. (Informant 6)

Another wish from some of the deceased persons was to be buried in the native country, in the same place as their parents. For the informants, it was very important to fulfil this wish, and they organized it very rapidly after the death, since the culture also prescribes a fast funeral.

I had promised her that she would be buried next to her father...she wanted to rest next to him, so I said I won't let you down. So, actually 24 hours after she passed away, I just went with her. We flew to Lebanon. (Informant 4)

Even with the other cultural or religious wishes that were mentioned, the families had neither any needs nor expectations that the staff should help them. They organized it within the family or with the help of persons in their social network.

No, not really no, because we have a lot. My father feels we have many good contacts who could help us, so we kind of didn't need that help from healthcare. (Informant 3)

### The Families’ Prerequisites for, and the Impact of, Informal Palliative Caregiving

Different families’ perquisites for palliative care in the home are dependent on their social situation, that is, accommodation and number of family members who can help in the caregiving. The burden on the individual informants varies, as well as on different family members in the same family.

#### Social Situation

The social situation was important for the burden on the individual family member. In some families, they were quite many who could share in the caregiving and, in these cases, the deceased person also wished for hospice care close to death. In one family, the deceased person died quite early in the palliative phase, so the person did not have to become bedridden with all the symptoms common in the final stage of a cancer disease and, thereby, not dependent on so much care and help from the family members.

It was nice for us; dad says this in retrospect, considering her illness, that she didn't have time to get to this part. You know, cancer patients get really, really bad, but she died because of her heart. (Informant 5)

However, in some families, the caregiving rested on mostly only one person because of the deceased person’s wish (see the sub-group wises and needs), or the couple have no children or other relatives living in Sweden, or because another family member also needed help. The burden for these persons was immense, especially when the deceased person even denied care from the specialized palliative home care team. After all, the feeling of responsibility towards the deceased person was superior to the own well-being and also, in some cases, caused an economic burden, which will be described later in the sub-group social support.

#### Emotions

The informants described a lot of feelings, especially negative ones. They talked a lot about denial, even though they were also aware of the seriousness of the situation.

But I lived in denial, even though I was aware of her condition. I didn't want to know. (Informant 7)

The informants were asked about expectations or any concerns before informal caregiving in the home with support from a specialized palliative home care team, and some expressed certain fears linked to the care of the deceased person.

He had a lot of edema. He was full of fluid, so he couldn't move. That was one of the things I was most worried about. The fact that he was quite big. I didn't know how we were going to lift him up and so on... mostly about if there was something urgent; that they wouldn't make it in time. (Informant 3)

Another strong feeling was the sense of responsibility for helping and caring for the deceased person, and this was also linked to a feeling of burden. In some of the narratives from the informants, an almost inhumane workload appears, and the sleep deficit was considerable during the home care period.

I wanted to take care of her and no one else; friends are friends, but she is my wife, and I have an obligation as a husband to her and take care of her, and I have decided that I want to take care of her for the last two months. (Informant 4)

Yes, so I thought it was a lot of work because it was so difficult to squeeze in a conversation as I needed to call the hospital because he needed me for food and toilet visits... I usually say that I got the first really good sleep after he passed away. So the sleep was really bad, he woke up very often. (Informant 2)

It was also quite common with feelings of guilt, some in connection with the denial and the reluctance to talk about the medical condition and, thereby, the missing opportunity to make an ending.

There are so many thoughts that come. Why didn't I say such and such, and it's a lot of why didn't I do that and remember memories from before and, so of course, we could have delved into it, but not when I'm not susceptible to such things in any way and can end it that way. (Informant 1)

Other reasons stated for feeling guilty included not having done enough to save the deceased person’s life or not discovering the symptoms earlier or asking for an examination of severe symptoms.

The night he died, it was so calm and so still. I fell into a deep sleep, almost like a coma. And first, he stopped breathing and I saw that he, should I have done cardiopulmonary resuscitation? Should I have done something else? Yes, I have a lot (guilt). (Informant 2)

Completely exhausted and sad that I didn't help my mother in the right way. I should have forced that doctor to examine her much earlier. (Informant 6)

However, there were also examples of informants that were satisfied with their contribution, as in the one in the quote below, with the feeling that they had done everything for the deceased.

I feel a clear conscience now that I have done it, that I have done everything. (Informant 4)

### The Staff Working in Accordance with a Palliative Care Approach

A central finding in the interviews was how satisfied the informants were with the staff, especially those who worked in the specialized palliative care; furthermore, it is clear from their narratives that the staff worked in accordance with the palliative care philosophy.

#### Social Support

The sub-code social support was divided into informative, emotional, and instrumental or practical support in accordance with House’s (1981) division in classes. Much of the given informative support is in connection with end-of -life (EOL) discussions. It was also clear that the EOL discussion was not only one separate conversation but several, at different times in the palliative process.

From the very beginning….They have informed us and supported us the whole time from the beginning; they told us what was going to happen, given that we were told that she would not live more than two months. (Informant 4)

What I also thought was very nice was when they saw that it was going downhill, because then a doctor came and talked to me. Then there were almost only hours left. I thought it was very nice then...it was hard, but it was great that it...well, those who are professionals, they look at the people and see that now it's getting closer; the breathing becomes different and the color becomes different too. And so, it was great that they said that. (Informant 1)

Although most of the informants were very pleased with the EOL discussions, they were also the source of what caused the most frustration and dissatisfaction with the care delivered. In one family, the hospital had failed to inform the deceased person that the treatment would be discontinued; therefore, the person waited to be called for continued treatment. The consequence was that the informant had to inform the whole family, as well as the deceased person.

They said ‘so we’re not going to continue treatment,’ and I said, ‘she’s not going to get any treatment for her tumor.’ He said no, and then I understood. Then I said, ‘you know, this hasn’t come up with my mother; she thinks she’s going to have the next appointment for treatment....’ He said, ‘oh, I apologize so much that there has been a misunderstanding....’ I don't know at all how I managed everything you know. You have to explain as if you’re a doctor to someone, your own mother. That is different for someone who is not a doctor. (Informant 5)

Another situation was when there was no clear decision to interrupt the treatment before the decision about home care and thus, no EOL discussion came about.

When she started the care at home, we did not have that conversation (EOL) because she would still have the injections, so that conversation was not relevant before she came home. (Informant 6)

Emotional support in palliative care is meant for both the patient and the family members. Almost all informants were very pleased with how the staff supported their sick family member or how they tried to.

I like to use my mother’s words; she was surprisingly satisfied, happy, as I said. She hated being a burden, but the times she asked for help, she felt that they weren’t there just to help and do their job, they are real heroes and that’s what she said, literally that sometimes they can be more humble, more emotional, than many friends can, because they stayed and really asked how are you and do you want us to stay extra, so all that little extra showed that they actually care about her well-being and she appreciated that very much. So, she was extremely satisfied. (Informant 7)

In some families, the emotional support for the informants was more or less absent, mainly because they did not live with the deceased person or they were too busy during the care-time; in addition, after the death, they did not have time or energy for bereavement support, even if almost all of them had been offered that. However, examples were also given when the informants had experienced very strong and important emotional support from the staff.

It was the beginning of AHC, then I had contact, and we talked about such serious things; it was great...A nurse and then, I also got papers that I could contact the counselor. I also met her. (Informant 1)

When they were about to leave, I remember they asked a few questions, and we got to the hall and then I had a really bad panic attack; a delayed reaction and then, I don't know if it was a doctor and a nurse maybe, or if it was two nurses, uh and they were very helpful then, very helpful. (Informant 2)

Concerning instrumental support, all informants were very pleased with all the practical help they received, and the facilities (shower chair, hospital bed etc.) that were delivered to their home. However, two informants had big problems with the benefit when caring for closely-related persons because the informant was a student and did not have an employment and, therefore, was not eligible for the benefit. In the other case, it was a problem because the 100 days were completed before the person died. This led to severe consequences for the informants. In the first case, there was no money to live on; in the other case, the informant had to return to work and at the same time, care for the deceased person.

Sent in my application, and I still haven't received a decision considering that I don't live in Sweden. And I study abroad and I have a high tuition fee, so what I have always done when I come back over the summer and in the winter and Easter, then I work as an assistant nurse at Uppsala university hospital and as an assistant nurse in home care at Uppsala…. So this summer went entirely to my father and so I had no income at all. You don't get a student grant during the summer. I couldn't even work. So I applied for it, but I haven't received it yet. (Informant 2)

I thought that the next-of-kin money would last the period with mother. An old person might have several periods, but I had misunderstood the whole thing. But I didn't have a hundred days left, but then I had used more than a third of 2018 when I thought mum would die of blood poisoning. So somehow, it became complicated… because leaving my mother was not an option for me. Unfortunately, it ended and then I had to start work and it was a nightmare; it was the worst time for me. (Informant 6)

#### Medical Treatment and Accessibility

The symptom relief was successful in most cases, and immediate assistance was available, both day and night.

Yes, so, there were no problems and at the end, they could come in the middle of the night too if we needed it; she had something like that the morphine went into a syringe, that's when they came yes, exactly that, and checked during the night too... Yes, among other things, yes she got as much as she needed. (Informant 1)

Nevertheless, informants also gave a few examples of non-appearances or investigations that were delayed because of a neglect of atypical symptoms in the end-of-life care.

To sort out what was not just related to the cancer, it was down prioritized. (Informant 6)

#### Competence and Attitude

The comments regarding the staffs’ competence and attitudes were mostly highly positive. A lot of these views are also apparent in quotes under other sub-codes in the findings. Common words the informants used to describe the staff were that they were professional and supportive and made the families feel secure with the care given in the home.

They were very professional; they were responsive, caring, pleasant and knowledgeable. They asked what do you need help with, but they gave her options. (Informant 5)

I always felt safe with the staff actually. Yes, I think, I received very good support, especially there (in the home) at the end. (Informant 3)

Some informants also gave examples of how well they thought their points of view on some care aspects were received by the staff.

Even if she has half an hour that she feels a little better, maybe you can take advantage and give her a better half hour by just visiting her a little more... they started doing that the next time when I was there. I saw that she and the nurse had music on and danced together, and it was an incredibly beautiful moment. So it helped, they listened to my point of view. (Informant 7)

### Palliative Care in a Seamless Care Chain

The care chain in a cancer course, when the disease has advanced already at time of diagnosis or progressed into a palliative phase, is usually hospital care, followed by specialized palliative home care or hospice care, or first specialized palliative home care and then hospice care in the final phase. However, the course is not so linear for all patients; the deceased persons in the interviews had different courses, although all of them were cared for in specialized palliative home care, but some died at the hospital, and some in the own home or at the hospice.

#### Hospital Care

After the EOL discussion, the transition from hospital to home care usually became very fast and smooth.

The doctor at the hospital had said that there were no more medicines; then, they stopped, and then AHC was very quickly connected and we got a lot of help. There was a physiotherapist, an occupational therapist and everyone who could, and yes, they came home and helped. (Informant 1)

However, when the deceased person was enrolled in the AHC without a clear EOL discussion, the transition became more problematic because the families waited for a call for continued treatment. Especially in one case, this resulted in a feeling of a poor coordination of care and caused an uncertainty as to which unit was responsible for the person’s care.

The most important thing was cooperation, coordination. I would have liked to have a documented plan, this breaking point conversation, e.g., who was responsible for it, who decided now...I probably would like to have had a little more concrete info of how they think because I think they were not always in sync. (Informant 6)

For another deceased person, it was necessary for her to be referred back to hospital care for treatment for an acute medical condition for some shorter periods; however, this person also passed away at the hospital.

She was in the hospital for a few days, and then she was at home maybe 4-5 days before we called and said that she can’t breathe; she has great difficulty breathing and then, she went into the hospital and then she was lying there, maybe I can’t remember if it was a week or a few days, and then she just passed away in her asleep. (Informant 5)

#### Specialized Palliative Home Care

In the families where the deceased person was only cared for by the AHC, from the EOL discussions to the death in the home, all informants expressed that they had received information about the alternative, hospice care. ‘*Yes, we were well informed about the options.*’ *(Informant 4).* Nevertheless, for them, this was not an alternative they had thought about because the deceased person wished it to be like this (see above), even though they did not see it as a good alternative.

We had received good information about the hospice, and we thought about it quite a lot, but it was better; he got what he wanted. (Informant 3)

#### Hospice Care

In the two cases where the deceased wished to move to the hospice after a period of care in the home, the transition was very fast (see the quote above). The informants were also very impressed by the reception at the hospice and all the facilities available to them as next-of-kin.

Yes, it was good; we got there in the middle of the night and they were prepared, and I got my own big room and there was even a couch you could pull out and sleep there in the same room. But I wanted to sit in one like that there armchair, sit next to her. The second night, her sister slept there too because then she also wanted to join, so she got the couch, and they even offered that there was a room somewhere else in case you wanted to go away and rest. There were also such things as coffee; you could go and get coffee ... Yes, and there was a refrigerator, and you could take things with you, and so that part was nothing I missed either. (Informant 1)

Even when the deceased person had been enrolled for hospice care, they could go back to home care again; however, this was not current with these two persons, but one went on leave the last two Sundays.

Everything flowed at the right time, so when she was home, it was appropriate to be there. And when she moved to the hospice, it was appropriate to be there. So that, for us, I think everyone was happy with it... for the last two weeks, we took her home on Sundays. (Informant 7)

## Discussion

This study is unique as it investigates preferences, expectations, and experiences on specialist palliative care from relatives of migrants. The main findings showed no support for the perception that the immigrant patient and their families had specific needs and wishes linked to cultural or religious issues, maybe with the exception of the strong wish for help with personal hygiene only from female staff. Concerning cultural issues or religious rituals, they did not expect the healthcare staff to have knowledge about that or be helpful with that kind of matter. So, the question is how much specific knowledge does the staff in specialized palliative care need to have about different cultures or religions in order to provide good palliative care? In our view, the most important, with all patients, is to work in accordance with the palliative care philosophy, that is, communicate with each patient and his or her family about their needs and preferences of care and to treat them accordingly. The concerns when caring for non-immigrant patients, in a palliative phase, in their home, as the staff expressed in earlier Swedish studies (Karlsson, 2008; Milberg et al., 2016; Torres et al., 2016), seem groundless in light of the findings reported in the present study. However, it probably relates to another situation when the patient and the family are newly arrived in Sweden and are not so well integrated as the families in the present study.

All family members were aware of the fatal prognosis and that the time remaining was restricted according to the informants; however, it was also apparent that they did not talk about it. In some cases, it was the deceased person who avoided the subject, but it was also the next-of-kin, almost to the same degree. The problem with this avoidance was that the informants came to regret this when it was too late, and it even caused feelings of guilt. The importance of the next-of-kin having sufficient time to be aware of the impending death is for them to have a better quality of life and mental health after the loss has been shown in earlier studies (Hauksdóttir et al., 2010; Valdimarsdóttir et al., 2004). In spite of this, it is obvious in the findings that awareness did not necessarily lead to an open communication in the family about the impending death. A study (Jonasson et al., 2011) that investigates the association between couples’ communication before the wife’s death and the widower’s feelings of guilt after the loss shows that men who thought that not everything had been brought to closure before their wives’ deaths had an increased risk of feeling guilt. Earlier studies have also shown that the next-of-kin’s feelings of guilt after the loss are associated with how they perceived that the healthcare provided end-of-life care (Holtslander et al., 2017; Ylitalo et al., 2008). However, in the present study, it is apparent that the informants, with a few exceptions, were very pleased with the care provided.

The burden on the informants was high and, in some cases, extremely high. The common denominator for them was that they did not share the caring responsibility with other family members and, in some cases, not even with the specialized palliative home care team. An earlier study by Milberg et al. (2003) showed the positive experiences of sharing the caring duties with the staff, both for the opportunity to participate in the care as much as wished, and for the psychological and/or physical relief for the next-of-kin to share the responsibility. It is important that the healthcare staff pay attention to the-next-of kin’s situation and recommend some kind of respite care or other form of assistance when needed to relieve the next-of-kin. However, in the present study, the informants were conscious of those opportunities, but they wanted to do whatever they could for the deceased person even when this was detrimental to their own health, well-being, and/or economy. This agrees with the concept of self-transcendence, to do something above oneself, which was something that Milberg and Strang (2003) also found in their study of what the next-of-kin found meaningful in palliative home care.

The findings challenge the taken-for-granted perceptions that caring for the migrant patients in specialized palliative home care is as meeting “the unknown” (Karlsson, 2008). Furthermore, shared knowledge in the family about the bad prognosis is not enough for an open communication in the family and indicates that healthcare professionals should encourage and support the family to communicate about this before it is too late.

## Method Discussion

The systematic text condensation method by Malterud (2012) was chosen for analyzing the material, and the method was well suited for the research question with a strong focus on meaning and responsibility in EOL care. Semi-structured individual interviews offered the possibility to communicate about emotional and sad topics and, at the same time, give individual confirmation to the informants. If the interview brought up strong feelings of sadness or negative memories, we had the opportunity to contact the social worker in the AHC-team. This happened only once with an informant, not because of negative feelings but due to a wish for a bereavement follow-up communication.

## Limitations

The study has some limitations worth noting. Concerning the data collection, interviews were conducted over a long time period. The reasons for this were that it was difficult to recruit next-of-kin to foreign-born persons and because the interviews had to be paused due to the COVID-19 pandemic, as the staff had a huge burden during that time. Thereafter, the interviews had to be carried out by telephone. However, no changes were noticed in the quality of the interviews due to the change in conducting the interviews by phone instead of face-to face, which is similar to previous studies by Mazzoni et al. (2019). The material was also very rich, despite having only a few informants. Another shortcoming in the study was that no wives were interviewed as they did not accept to participate.

The qualitative study design made it possible to deepen the knowledge about the studied phenomenon and identify different perspectives to try to understand the informants’ views, instead of explaining results that can be generalized to a wider population (Patton, 2015). We consider our findings to be transferable to next-of-kin of foreign-born patients in a late palliative phase setting, who are cared for in specialized palliative care, especially when the family has lived in Sweden for a long time and are well integrated.

## Appendix A

### Interview Guide

• What country was the diseased born in and when was the immigration to Sweden
• Your relation to the diseased
• Tell me about expectations and concerns infront of caring the diseased in the home with help of the palliative home care team
  - The diseased (him/her selve)
  - Different next-of-kin
• Which next-of-kins where involved in the care
• Tell me about how you experienced that the care function when the diseased was cared for in the home
  - Communication, end-of-life discussions, the deceased’s awareness about the limited time, the need of an interpreter
  - The responsibility
  - The support to the diseased (from the staff, other family members, others)
  - The support to the informant (from staff, other family members, others), the benefit when caring for closely-related persons, bereavement support
  - Became the care as expected
  - Hade the diseased or family specific wishes (religious or cultural)
  - Did you miss something
  - If applicable, tell me about the care at hospice or other care institution (if the diseased did not died at home)
• Tell me about your knowledge about other care alternative than palliative home care
• Something more you will tell me about your experience of the care in the palliative phase of the disease
